# Evaluation of Parity Effect on Characteristics and Minerals in Buffalo (Bubalus Bubalis) Colostrum and Mature Milk

**DOI:** 10.3390/foods12061321

**Published:** 2023-03-20

**Authors:** Zhigao An, Gan Luo, Shanshan Gao, Xinxin Zhang, Chao Chen, Zhiqiu Yao, Junwei Zhao, Haimiao Lv, Kaifeng Niu, Pei Nie, Liguo Yang

**Affiliations:** 1 National Center for International Research on Animal Genetics, Breeding and Reproduction (NCIRAGBR), Ministry of Science and Technology of the People’s Republic of China, Huazhong Agricultural University, Wuhan 430070, China; 2 Key Laboratory of Animal Genetics, Breeding and Reproduction, Ministry of Education, College of Animal Science and Technology, Huazhong Agricultural University, Wuhan 430070, China; 3 International Joint Research Centre for Animal Genetics, Breeding and Reproduction (IJRCAGBR), Huazhong Agricultural University, Wuhan 430070, China; 4 Hubei Province’s Engineering Research Center in Buffalo Breeding and Products, Wuhan 430070, China

**Keywords:** buffaloes, colostrum, mature milk, parity

## Abstract

Colostrum is a vital performance for buffaloes and potentially functional foods in the future. Therefore, this study aimed to evaluate the difference between the parity of buffalo colostrum and mature milk. Twenty pregnant buffaloes (primiparous = 10; multiparous = 10) were assigned to the same diet prepartum and milking routine postpartum. Calves were separated from the dams immediately after birth and colostrum was harvested within 2 h, whilst mature milk was harvested at 7 days postpartum. The colostrum was analyzed for immunoglobulin G and milk composition as the mature milk. The results showed that there was a higher level of protein, solid not fat, and milk urea nitrogen (*p* < 0.05), with a tendency for higher total solids (*p* = 0.08) in primiparous buffaloes’ colostrum compared with multiparous. No parity effect was observed in colostrum immunoglobulin G, fat, lactose, and yields of colostrum and composition (*p* > 0.05). There was no difference in mature milk composition and yield by parity affected (*p* > 0.05). Compared with mature milk composition, colostrum had a higher content protein, total solids, solid not fat, and milk urea nitrogen (*p* < 0.05); however, fat and lactose were lower than that of mature milk (*p* < 0.05). For minerals, multiparous buffaloes’ colostrum had a higher concentration of Fe (*p* = 0.05), while the mature milk had higher concentrations of K and P compared with primiparous. Buffalo colostrum had higher concentrations of Na, Mg, Co, Fe, and K with a lower concentration of Ca relative to mature milk (*p* < 0.05). It was observed that parity affected colostrum characteristics rather than mature milk and caused subtle variations in minerals in colostrum and mature milk of buffaloes. As lactation proceeded, both milk composition and minerals in the milk changed drastically.

## 1. Introduction

Colostrum is the preliminary milk secreted after dams’ parturition. In terms of composition, colostrum has a content of higher milk fat and protein relative to mature milk [1]. Therefore, colostrum is more dense and yellow than mature milk [2]. Among the colostrum components, the most significant difference is milk protein and the difference in proteome between buffalo colostrum and mature milk has been reported [3]. Unlike mature milk, which contains casein as the main protein fraction, the main protein fraction in colostrum is immunoglobulins. Colostrum immunoglobulins provide passive immunity to calves in the first few weeks. The main bovine immunoglobulin concentration ranges from 50 mg/mL to 150 mg/mL, of which approximately 85% to 95% is immunoglobulin G (IgG) [4]. Although colostrum cannot be consumed as regular milk, it can be consumed as a special functional food by special populations of weakened immune function (elderly subjects and neonates) [5]. In addition, colostrum has other functions, such as to reduce intestinal permeability [6], regulate intestinal immunity [7], and reduce inflammation [8]. As lactation proceeds, colostrum is gradually converted to mature milk. After five days postpartum, the milk produced by the dams is mature milk, which is the primary production value [9].

Minerals have an essential function in physiology as a vital section of colostrum and mature milk composition such as constituting tissues and organs, maintaining osmotic pressure and acid-base balance, and catalyzing regulation [10]. The mineral content of colostrum is relevant to the mineral status of newborn calves. Compared to mature milk, colostrum has a higher concentration of minerals and therefore has a unique role in the growth and development of calves [11]. Because mature milk is rich in minerals, it is an essential source of the daily intake of minerals for humans. The mineral content of mature milk is also related to milk quality and physicochemical indicators [12]. Although studies have shown changes in the minerals of Holstein cows’ milk [13,14], parity as an influencing factor in buffaloes has not been studied.

Buffaloes, the second largest dairy livestock, account for 14.4% of the world’s milk production due to their outstanding milk quality and productivity [15]. Buffalo milk has higher total solids, fat, and minerals than Holstein cows’ milk [16]. Although several studies have measured the composition of buffalo colostrum, few reports have been made on colostrum and composition yield [17,18]. Buffalo milk meets the nutritional requirements as an essential source of milk. Buffalo milk products, such as mozzarella, yogurt, and ghee, are also popular. Several factors such as breed, parity, calf sex, calving month, and health status are related to colostrum production [19]. Additionally, parity as a vital factor affecting colostrum characteristics has been studied in Holstein cows [20,21] but has not been mentioned in buffaloes. On the other hand, the vest metabolic differences in buffaloes during early lactation resulted in content differences. The study of composition changes cannot be neglected due to the dynamic characteristics and correlation between colostrum and mature milk [22]. 

The purpose of this study was to analyze the distinction between colostrum composition and subsequent mature milk and to evaluate the influence of parity and lactation time on its characteristics and minerals.

## 2. Materials and Methods

All colostrum and mature milk samples were collected at a commercial buffalo ranch (Jinniu Animal Husbandry Co., Ltd., Jingmen, China) in September 2022. All buffaloes were properly cared for and approved by the Animal Ethics Committee of HZAU (HZAUBU–2020–0004).

### 2.1. Dams’ Management and Sample Collection

Twenty buffaloes (primiparous = 10; multiparous = 10) were assigned in the same enclosure and provided total mixed ration (TMR) twice daily, containing corn silage, peanut vine, straw, and mixed concentrate. Nutrient contents consisted of crude protein 9.65%, ether extract 2.36%, neutral detergent fiber 54.57%, and ash 7.17%. The nutrient requirements to meet dry buffaloes are based on the report of Bartocci et al. [23]. Watching the dams’ behavior when close to the calving time and delivery assistance with the calves’ arrangement were observed. Calves were separated from the dams immediately after calving to avoid teat sucking, and dams were placed in the milking parlor to harvest the colostrum within 2 h. The colostrum was collected after manual stimulation for 30 s, including pre-dipping, forest ripping (removal of 3 streams of colostrum from each teat), and placed in the milking machine (Asahi Bronte Machinery Co., Ltd., Zibo, China). After colostrum harvest, the weight was measured using an electronic scale, transferred without loss to a measuring cup for volume determination, and two equal samples (50 mL) were collected for composition analyses. Dams were moved into separate lactation pens that provided the same TMR twice daily, containing corn silage, peanut vine, and mixed concentrate. Nutrient contents included crude protein 12.36%, ether extract 3.04%, neutral detergent fiber 38.14%, and ash 5.26%. These dietary nutrients fulfill the milking buffaloes’ nutrient requirements [23]. Mature milk samples (50 mL) were harvested in the same procedure as colostrum at 7 days postpartum based on the previous report [9]. All calves were born without any calving complications. Potassium dichromate was added as a preservative to the first samples of colostrum and mature milk and stored at 4 °C until milk composition was analyzed, and the second was stored at −80 °C for IgG and minerals analysis.

### 2.2. Composition and Minerals Analyses

Samples (50 mL) of colostrum and mature milk stored at 4 °C were sent to the Dairy Herd Improvement of Hubei province for milk composition testing using a milk composition analyzer (CombiFoss FT+, Shanghai Jinmai Instrument Equipment Co., Ltd., Shanghai, China) to analyze fat, protein, lactose, total solids (TSs), milk urea nitrogen (MUN), and solid not fat (SNF). Due to the high viscosity of colostrum, it was mixed with double-distilled water in equal volumes prior to milk composition testing. After the test, the milk content was doubled to obtain the final result. Colostrum IgG assay was performed using a commercial enzyme-linked immunosorbent assay (ELISA) kit (catalog number RX1600804 B, Quanzhou Ruixin Biological Technology Co., Ltd., Quanzhou, China), following instructions according to the method of the previous study [24].

Minerals including calcium (Ca), sodium (Na), potassium (K), magnesium (Mg), cobalt (Co), and iron (Fe) in colostrum and mature milk were determined using flame atomic absorption spectroscopy (Agilent 240 FS, Agilent Technologies, Inc., Mulgrave, VIC, Australia), as was reported by Mi et al. [25]. Briefly, 0.5 g colostrum and mature milk were digested by adding 5 mL of 65% nitric acid and 2 mL of hydrogen peroxide before heating. The resulting ash was dissolved in 5% nitric acid and made to 50 mL for analysis. Phosphorus (P) was determined using a colorimetric method. Next, 0.5 g of the milk sample and 20 mL of nitric acid were added into a Kjeldahl flask and boiled. A total of 10 mL of 70% perchloric acid was added and boiled until colorless, after which the volume was adjusted to 50 mL with double-distilled water. The nitrated sample was added to ammonium molybdovanadate for colorimetric determination at 420 nm.

### 2.3. Statistical Analyses

One-way analysis of variance (ANOVA) was used to analyze the IgG concentration and yield. All data except IgG were analyzed using PROC MIXED procedure of SAS 9.4 (SAS Institute Inc., Cary, CA, USA). The model included parity, milking, the interaction between parity and milking as fixed effects, and random effects of buffaloes.

(1) $$Y_{ijk}=\mu +M_{i}+P_{j}+MP_{ij}+\varepsilon_{ijk}$$

where *Y_ijk_* = the *k*th observation in the *i*th milking and *j*th parity; *µ* = overall mean; *M_i_* = fixed effect of the *i*th milking; *P_j_* = fixed effect of the *j*th parity; *MP_ij_* = fixed effect of the interaction between the *i*th milking and *j*th parity; *ε_ijk_* = random error in the *k*th observation in the *i*th milking and *j*th parity. The results are reported as least squares means and standard errors of means (SEMs). The effect was significant at *p* ≤ 0.05, whereas trends were considered when 0.05 < *p* ≤ 0.10.

## 3. Results and Discussion

### 3.1. Differences in Colostrum Composition between Different Parities

The IgG concentrations in primiparous and multiparous buffalo colostrum were 35.25 mg/mL and 39.85 mg/mL (Table 1), respectively. There was no previous study on the effect of parity in buffalo colostrum IgG. Gulliksen et al. [26] confirmed that parity was associated with colostrum IgG concentration in Holstein cows; however, this was not found in the current study (*p* = 0.24). In the current study, primiparous buffaloes had higher SNF (*p* = 0.03) and tendency of TS (*p* = 0.08) than multiparous buffaloes because of the lack of a parity affect in milk fat (*p* = 0.81), which was similar to previous studies [27,28]. The colostrum protein of primiparous buffaloes was 20.77% higher than 17.69% in multiparous (*p* = 0.02). Similarly, in the study of Soufleri et al. [29], parity significantly affected colostrum protein in Holstein cows. There was no difference in IgG between different parities; therefore, other protein components were higher, since colostrum protein accounted for approximately 50% of the IgG [30]. Similarly, differences in nitrogen excretion are also reflected in MUN, with primiparous buffaloes having higher MUN relative to multiparous buffaloes (*p* = 0.03). This accounts for the difference in colostrum protein. Regarding colostrum yield, multiparous buffaloes show numerically higher colostrum yield than primiparous (*p* > 0.05). In Holstein cows, colostrum yield was 27.5–37.5% higher in multiparous than primiparous due to sufficient mammary gland development [31], whereas, in buffaloes, the value is 12.27%, albeit merely in numerical (*p* = 0.75). 

### 3.2. Differences in Mature Milk Composition between Different Parities

Table 2 indicates the distinction in mature milk between different parities. There was no difference between the mature milk in primiparous and multiparous buffaloes at 7 days postpartum. Two research studies consisting of 220 and 248 buffaloes found that parity did not affect milk yield, respectively [32,33]. Similarly, Poudel et al. [34] reported an identical conclusion when investigating livestock farmers. However, some studies on buffaloes have been reported as controversial. Verma et al. [35] and Alkhateeb et al. [36] studied 176 and 72 buffaloes and found that multiparous buffalo have a higher milk yield, respectively. Although it has been shown that milk production in Holstein cows’ increases with parity based on improved weight gain and mammary gland development, the effect of parity on buffaloes’ milk yield requires further study. Moreover, the differences caused by parity in milk composition are often inconsistent. Alkhateeb, Ibrahim, and Al-Anbari [36] showed that parity affected milk protein, while a study of 94 buffaloes showed no difference in milk protein [37]. Since milk composition is related to milk yield, it is more pertinent to examine milk composition yield; however, few studies have reported milk component yield.

### 3.3. Characteristic of Colostrum to Mature Milk in Buffaloes

The difference in milk composition and yield between buffalo colostrum and mature milk is shown in Table 3. Parity did not affect milk composition and yield transformation from colostrum to mature milk (*p* > 0.05). Besides SNF yield, milking time induced differences in all compositions were detected (*p* < 0.05). As lactation progressed, the protein content changed from 19.23% in colostrum to 5.54% in mature milk with a sharp drop (*p* < 0.01), comparable to previous studies on buffaloes [9,38,39]. The cessation of immunoglobulins secretion after calving induces a protein decrease in the maturing milk based on immunoglobulins, comprising 70–80% of the total protein in colostrum [40]. The colostrum lactose of buffaloes was 2.55%, similar to the previous reports [9,41]. On the contrary, Mudgal et al. [42] showed that the lactose in colostrum was 4.38–6.31%, which was nearly 2–3 times that as reported here; however, there was 5.16–6.47% colostrum protein in the same report, and it is some distance from the present study. Lactose was increased in mature milk compared to colostrum (*p* < 0.01). Milk lactose account for about 50% of the osmotic pressure of milk [43]; moreover, it causes water to move from the cytoplasm of the mammary epithelial cells into the milk. Thus, low levels of lactose result in the production of extremely viscous milk with high solid content due to a lack of lactose [44]. This is similar to the patterns of lactose and their increase from colostrum to mature milk in buffaloes [45], bovine [1], and yak [25]. Fat was lower in colostrum than mature milk in this experiment (*p* < 0.05), which is consistent with previous studies [38,45]. It has been reported that the colostrum fat was 8.04% (higher than 3.90% five days later) to support the thermoregulation and rapid metabolism of newborn Holstein calves [2,46]. Despite an increase in milk yield with milking, colostrum protein production was higher than mature milk due to its excellent protein percentage relative to mature milk (19.23% versus 5.54%). There was no difference in the yield of SNF between colostrum and mature milk (*p* = 0.64), while the yield of TS was higher in mature milk (*p* < 0.01), which was due to an increase in milk fat. Bizarrely, it was mentioned in the report of Bondoc and Ramos [47] that the colostrum yield was 4.94 ± 0.30 kg; however, the result of this study was 1.73 ± 0.82 kg, and another study was similar to the present study [45].

### 3.4. Differences in Colostrum Minerals between Different Parities

The parity effect on the colostrum minerals is shown in Table 4. Fe concentration was higher in the multiparous buffaloes than primiparous (14.04 mg/kg versus 7.94 mg/kg) (*p* = 0.05), although there was no difference in Fe yield. In the buffalo colostrum, the Fe content varied and was higher than the 2.36 mg/100 g reported elsewhere [9,39]. Moretti et al. [48] recommended a decreasing trend in total iron binding capacity and transferrin concentration in the colostrum of primiparous Holstein cows relative to multiparous ones despite no distinction in Fe concentration. In the present study, Ca and Mg concentrations in the colostrum of primiparous and multiparous buffaloes were 5.83 g/100 g versus 6.22 g/100 g and 1.92 g/100 g versus 1.86 g/100 g, respectively, which was comparable to the previous study [49]. Meanwhile, Na and K concentrations were 10.82 g/100 g versus 8.84 g/100 g and 12.19 g/100 g versus 12.70 g/100 g in the colostrum of primiparous and multiparous buffaloes, respectively, which were lower than in the previous study [39]. In this experiment, Ca, Na, P, K, Mg, and Fe concentrations were higher than those reported by El-Fattah, Alaa, Abd Rabo, EL-Dieb, and El-Kashef [9], for whom the mean values of concentrations were 279.60 mg/100 g, 150.50 mg/100 g, 58.00 mg/100 g, 107.00 mg/100 g, 35.52 mg/100 g, and 2.36 mg/100 g, respectively, in contrast to the present study. 

### 3.5. Differences in Mature Milk Minerals between Different Parities

Table 5 shows the parity variations in minerals in mature milk. The P concentration in multiparous buffaloes was 12.58 g/100 g, higher than 10.24 g/100 g in primiparous (*p* = 0.04). This increase was associated with higher growth hormone activity, enhanced P uptake in the gut, and P reuptake in the kidney. However, it has been reported that blood P concentration was lower in multiparous Holstein cows [50,51]. Additionally, P concentration showed no significant correlation between blood and milk, and further discussion is needed to investigate it [14]. Likewise, K concentration in multiparous buffaloes also exhibited variations compared with primiparous (18.41 g/100 g versus 16.20 g/100 g) (*p* = 0.05). In contrast, Stocco et al. [52] confirmed that the parity did not affect the K concentration in buffaloes. Although there was no distinction in Na concentration, there was a tendency for Na yield to be higher in primiparous buffaloes than in multiparous ones in present study (*p* = 0.09). It has been proven that the Na concentration was higher in the multiparous buffaloes than in the primiparous buffaloes, based on an increase in milk production [53]. Na and K are involved in preserving the osmotic stability between blood and milk; however, the impact of parity on them is indistinct [54].

### 3.6. Minerals of Colostrum to Mature Milk in Buffaloes

Changes in minerals in buffalo colostrum to mature milk are shown in Table 6. Ca concentration increased from 6.02 g/100 g in colostrum to 10.05 g/100 g in mature milk (*p* < 0.01); however, P concentration showed no distinction (10.12 g/100 g verses 11.41 g/100 g) in the present study (*p* = 0.14). The high Ca concentration in mature milk makes milk a good source of Ca supplements for human intake or for calves’ growth. However, the steep depletion of Ca makes it possible for cows to experience milk fever (parturient hypocalcemia), which leads to an increased risk of other diseases [55]. It has been shown that higher concentrations of minerals could increase the total antioxidant capacity of calves, and that mineral concentrations tend to decrease again as lactation proceeds, as has been proven in various lactating animals [14,25,56]. In the present experiment, Mg and Fe concentrations decreased from 1.89 g/100 g and 10.99 mg/kg in colostrum to 1.42 g/100 g and 4.69 mg/kg in mature milk (*p* < 0.01), respectively. Co present in milk, mainly in the form of cobalamin (vitamin B12), is the primary source of vitamin B12. Cobalamin as a coenzyme plays an important role in ruminant energy metabolism [57]. The cobalt concentration decreased from 4.47 mg/kg to 3.11 mg/kg, which is higher than a previous report [58]. 

## 4. Conclusions

Parity affected the composition of the colostrum of buffaloes, which was demonstrated by primiparous buffaloes having higher protein, SNF, and MUN compared to multiparous ones, while no influences in mature milk were found. Although no influence was noted in mature milk, the fat and lactose content increased while protein, TS, SNF, and MUN were decreased in the transition from colostrum to mature milk. In terms of minerals, parity affected Fe concentration in colostrum and P and K concentration in mature milk. Colostrum had higher Mg, Co, and Fe, while lower Ca and K concentrations relative to mature milk. As lactation proceeds, there is a massive distinction in milk composition and minerals between colostrum and mature milk almost without any affect due to parity.

## Figures and Tables

**Table 1:** Differences in colostrum composition and yields between primiparous (n = 10) and multiparous (n = 10) buffaloes.

Item	Parity ^1^		SEM	*p*-Value
	Primiparous	Multiparous		
Composition				
IgG (mg/mL)	35.25	39.85	2.70	0.24
Fat (%)	4.87	5.10	0.30	0.81
Protein (%)	20.77	17.69	0.91	0.02
Lactose (%)	2.34	2.75	0.12	0.16
TS (%)	31.55	28.59	0.99	0.08
MUN (mg/dL)	132.70	112.54	6.42	0.03
SNF (%)	25.15	22.12	0.93	0.03
Yield				
Colostrum (kg)	1.63	1.83	0.18	0.75
IgG (g)	56.92	75.27	9.07	0.17
Fat (g)	7.82	9.33	1.12	0.74
Protein (g)	32.09	31.36	3.00	0.89
Lactose (g)	4.04	5.13	0.58	0.71
TS (g)	49.53	51.22	4.94	0.89
SNF (g)	39.36	39.38	3.78	0.99

^1^ Values represent means: primiparous buffaloes (n = 10), multiparous buffaloes (n = 10).

**Table 2:** Differences of mature milk composition and yields between primiparous (n = 10) and multiparous (n = 10) buffaloes.

Item	Parity ^1^		SEM	*p*-Value
	Primiparous	Multiparous		
Composition				
Fat (%)	6.85	7.68	0.59	0.39
Protein (%)	5.37	5.71	0.16	0.78
Lactose (%)	4.21	4.34	0.17	0.65
TS (%)	17.39	18.74	0.68	0.42
MUN (mg/dL)	28.20	31.10	1.29	0.75
SNF (%)	10.40	10.99	0.21	0.65
Yield				
Mature milk (kg)	4.28	3.47	0.39	0.20
Fat (g)	29.54	24.72	2.93	0.29
Protein (g)	22.28	19.64	1.99	0.62
Lactose (g)	18.29	15.44	1.93	0.33
TS (g)	73.99	63.18	6.85	0.38
SNF (g)	43.99	38.22	4.15	0.48

^1^ Values represent means of those for primiparous buffaloes (n = 10) and multiparous buffaloes (n = 10).

**Table 3:** Least squares means and 95% CI for composition and yields in colostrum and mature milk with different parities and milking time (n = 20).

Item	Milking		SEM	*p*-Value ^1^		
	Colostrum	Mature Milk		Parity	Milking Time	P × M
Composition						
Fat (%)	4.99	7.26	0.47	0.30	0.01	0.72
	4.36–5.62	6.04–8.49				
Protein (%)	19.23	5.54	0.62	0.16	<0.01	0.05
	17.32–21.13	5.21–5.87				
Lactose (%)	2.55	4.28	0.14	0.28	<0.01	0.38
	2.31–2.79	3.92–4.63				
TS (%)	30.07	18.06	0.83	0.48	<0.01	0.10
	27.99–32.15	16.64–19.49				
MUN (mg/dL)	122.62	29.60	4.44	0.20	<0.01	0.07
	109.18–136.06	26.90–32.30				
SNF (%)	23.63	10.70	0.65	0.19	<0.01	0.07
	21.68–25.59	10.25–11.14				
Yield						
Milk (kg)	1.73	3.88	0.31	0.57	<0.01	0.13
	1.34–2.11	3.05–4.70				
Fat (g)	8.58	27.13	2.24	0.67	<0.01	0.21
	6.23–10.92	20.99–33.27				
Protein (g)	31.72	20.96	2.60	0.72	<0.01	0.68
	25.44–38.00	16.79–25.14				
Lactose (g)	4.59	16.87	1.44	0.71	<0.01	0.26
	3.37–5.80	12.82–20.92				
TS (g)	50.37	68.59	6.07	0.68	<0.01	0.23
	40.03–60.71	54.24–82.93				
SNF (g)	39.39	41.10	4.04	0.70	0.64	0.43
	31.49–47.30	32.42–49.78				

^1^ P × M means affect between primiparous and multiparous buffaloes within milking time.

**Table 4:** Differences in colostrum mineral concentrations and yields between primiparous (n = 10) and multiparous (n = 10) buffaloes.

Item	Parity ^1^		SEM	*p*-Value
	Primiparous	Multiparous		
Concentration				
Ca (g/100 g)	5.83	6.22	0.48	0.68
Na (g/100 g)	10.82	8.84	0.56	0.58
P (g/100 g)	10.12	10.11	0.58	0.99
K (g/100 g)	12.19	12.70	0.43	0.64
Mg (g/100 g)	1.92	1.86	0.07	0.60
Co (mg/kg)	4.94	4.00	0.30	0.14
Fe (mg/kg)	7.94	14.04	1.96	0.05
Yield				
Ca (g)	0.92	1.11	0.13	0.76
Na (g)	1.50	1.84	0.17	0.72
P (g)	1.43	1.84	0.18	0.61
K (g)	1.83	2.30	0.23	0.68
Mg (g)	0.27	0.34	0.03	0.45
Co (mg)	7.56	6.97	0.91	0.86
Fe (mg)	13.13	23.15	2.98	0.14

^1^ Values represent means: primiparous buffaloes (n = 10), multiparous buffaloes (n = 10).

**Table 5:** Differences of mature milk mineral concentrations and yields between primiparous (n = 10) and multiparous (n = 10) buffaloes.

Item	Parity ^1^		SEM	*p*-Value
	Primiparous	Multiparous		
Concentration				
Ca (g/100 g)	9.53	10.57	0.44	0.27
Na (g/100 g)	9.94	7.90	1.17	0.28
P (g/100 g)	10.24	12.58	0.56	0.04
K (g/100 g)	16.20	18.41	0.68	0.05
Mg (g/100 g)	0.14	0.15	0.04	0.31
Co (mg/kg)	3.18	3.03	0.33	0.81
Fe (mg/kg)	3.74	5.64	0.99	0.53
Yield				
Ca (g)	4.09	3.63	0.42	0.46
Na (g)	4.18	2.58	0.62	0.09
P (g)	4.42	4.54	0.52	0.89
K (g)	6.94	6.54	0.76	0.74
Mg (g)	0.56	0.51	0.05	0.60
Co (mg)	14.56	10.70	2.12	0.25
Fe (mg)	13.74	18.60	3.62	0.46

^1^ Values represent means of those for primiparous buffaloes (n = 10) and multiparous buffaloes (n = 10).

**Table 6:** Least squares means and 95% CI for mineral concentrations and yields in colostrum and mature milk mineral with different parities and milking time (n = 20).

Item	Milking		SEM	*p*-Value ^1^		
	Colostrum	Mature milk		Parity	Milking Time	P × M
Concentration						
Ca (g/100 g)	6.02	10.05	0.46	0.28	<0.01	0.64
	5.02–7.03	9.14–10.96				
Na (g/100 g)	10.30	8.92	0.92	0.24	0.32	0.72
	9.14–11.46	6.46–11.38				
P (g/100 g)	10.12	11.41	0.56	0.13	0.14	0.17
	8.90–11.33	10.23–12.59				
K (g/100 g)	12.45	17.30	0.55	0.07	<0.01	0.33
	11.55–13.34	15.87–18.73				
Mg (g/100 g)	1.89	1.42	0.06	0.77	<0.01	0.18
	1.74–2.04	1.33–1.50				
Co (mg/kg)	4.47	3.11	0.31	0.31	<0.01	0.26
	3.84–5.10	2.42–3.79				
Fe (mg/kg)	10.99	4.69	1.50	0.09	0.01	0.32
	6.88–15.11	2.62–6.77				
Yield						
Ca (g)	1.01	3.86	0.31	0.79	<0.01	0.39
	0.75–1.28	2.99–4.73				
Na (g)	1.67	3.38	0.46	0.36	0.01	0.13
	1.31–2.04	2.05–4.70				
P (g)	1.64	4.48	0.40	0.68	<0.01	0.77
	1.26–2.01	3.40–5.57				
K (g)	2.07	6.74	0.57	0.96	<0.01	0.57
	1.59–2.54	5.15–8.32				
Mg (g)	0.31	0.53	0.04	0.89	<0.01	0.22
	0.24–0.37	0.43–0.64				
Co (mg)	7.26	12.63	1.64	0.43	0.01	0.35
	5.36–9.17	8.20–17.07				
Fe (mg)	18.14	16.17	3.28	0.21	0.55	0.44
	11.89–24.38	8.59–23.75				

^1^ Values represent means of those for primiparous buffaloes (n = 10) and multiparous buffaloes (n = 10).

## Data Availability

The original contributions presented in the study are included in the article; further inquiries can be directed to the corresponding authors.
